# Tri-specific killer engager: unleashing multi-synergic power against cancer

**DOI:** 10.37349/etat.2024.00227

**Published:** 2024-04-25

**Authors:** Peeranut Winidmanokul, Aussara Panya, Seiji Okada

**Affiliations:** Istituto Nazionale Tumori-IRCCS-Fondazione G. Pascale, Italy; ^1^Department of Biology, Faculty of Science, Chiang Mai University, Chiang Mai 50200, Thailand; ^2^Division of Hematopoiesis, Joint Research Center for Human Retrovirus Infection, Kumamoto University, Kumamoto 860-0811, Japan; ^3^Cell Engineering for Cancer Therapy Research Group, Chiang Mai University, Chiang Mai 50200, Thailand

**Keywords:** Tri-specific killer engager, natural killer cell, immunotherapy, cancer

## Abstract

Cancer continues to be a global health concern, necessitating innovative solutions for treatment. Tri-specific killer engagers (TriKEs) have emerged as a promising class of immunotherapeutic agents, offering a multifaceted approach to cancer treatment. TriKEs simultaneously engage and activate natural killer (NK) cells while specifically targeting cancer cells, representing an outstanding advancement in immunotherapy. This review explores the generation and mechanisms of TriKEs, highlighting their advantages over other immunotherapies and discussing their potential impact on clinical trials and cancer treatment. TriKEs are composed of three distinct domains, primarily antibody-derived building blocks, linked together by short amino acid sequences. They incorporate critical elements, anti-cluster of differentiation 16 (CD16) and interleukin-15 (IL-15), which activate and enhance NK cell function, together with specific antibody to target each cancer. TriKEs exhibit remarkable potential in preclinical and early clinical studies across various cancer types, making them a versatile tool in cancer immunotherapy. Comparative analyses with other immunotherapies, such as chimeric antigen receptor-T (CAR-T) cell therapy, immune checkpoint inhibitors (ICIs), cytokine therapies, and monoclonal antibodies (mAbs), reveal the unique advantages of TriKEs. They offer a safer pathway for immunotherapy by targeting cancer cells without hyperactivating T cells, reducing off-target effects and complications. The future of TriKEs involves addressing challenges related to dosing, tumor-associated antigen (TAA) expression, and NK cell suppression. Researchers are exploring innovative dosing strategies, enhancing specificity through tumor-specific antigens (TSAs), and combining TriKEs with other therapies for increased efficacy.

## Introduction

Recently, cancer has become a widespread occurrence worldwide presenting a challenge due to the absence of an effective solution [1, 2]. It exhibits a continuous increase in the USA and UK, especially in various types such as blood cancers, prostate cancer, and breast cancer [2–4]. Although some cancer reports indicate a decline in mortality due to advanced treatments, the estimated number of cases continues to rise annually in the USA, UK, and Asia. This raising is attributed to numerous risks associated with everyday life that persist without cessation [2–6].

Tri-specific killer engagers (TriKEs) are a novel class of immunotherapeutic agents that have emerged in recent years, holding great promise in the field of cancer treatment. TriKEs were engineered under the determination of the limitations of existing therapy. This fusion protein can both engage and activate natural killer (NK) cells, thereby sensitizing cancer cells for an immune attack [7–9]. TriKEs represent an outstanding advancement in immunotherapy, particularly in addressing the challenges of NK cell-based therapies. In 2016, Vallera et al. [7] introduced the concept of TriKEs by ingeniously incorporating interleukin-15 (IL-15) into the existing cluster of differentiation 16 (CD16) × CD33 bi-specific killer engager (BiKE), creating what is similar to a tri-specific antibody (TsAb). This innovation was a response to the critical issues of NK cell persistence and limited proliferation rates observed *in vivo*, paving the way for a more effective approach to cancer immunotherapy [8].

What sets TriKEs apart from other immunotherapies is their ability to simultaneously serve multiple functions while maintaining lower-to-no complications in NK cell function, illustrated by *in vivo* studies [9–11] and clinical studies [12]. Furthermore, their off-the-shelf nature brings the promise of convenience and accessibility to a broader range of patients. In recent years, TriKEs have demonstrated remarkable potential against various cancer types, indicating their promise as effective treatments shortly [7–12]. This review article delves into the generation and mechanisms of TriKEs, explore the advantages they offer over other immunotherapies, and discuss the future direction of TriKEs in cancer immunotherapy, including their potential impact on clinical trials and the treatment landscape.

## TriKE

TriKEs represent a notable advancement in the field of immunotherapy, offering a unique approach to combat cancer by orchestrating the innate immune system’s potent cytotoxic capabilities. TriKEs have the distinct ability to simultaneously target both cancer cells and NK cells, effectively creating a bridge between enhanced NK cell function and highly targeted cancer cell destruction [7]. In its earliest iterations, TriKEs were composed solely of single-chain fragment variables (scFvs) binding domains. These scFvs served as the essential components for recognizing specific antigens presented on the surface of cancer cells [13–16]. However, recent developments have elevated TriKE technology by replacing the major binding domain, typically an anti-CD16 scFv, with single-domain antibodies (sdAbs). These sdAbs are derived from the heavy chains of camelids, such as camels and llamas, and have demonstrated higher binding efficacy when compared to scFvs. This strategic transition to sdAbs not only enhances the binding affinity of TriKEs but also contributes to their overall effectiveness in targeting cancer cells with remarkable precision [17–20].

The action of TriKEs is a testament to their unique tri-specificity. These engineered molecules are designed to bind simultaneously to three key components: the target antigen on cancer cells, CD16 receptors on NK cells, and IL-15 receptors, an additional domain that promotes the persistence and activation of NK cells. This multifaceted approach is at the heart of TriKEs’ efficacy [7, 8, 20]. The binding to cancer cells is typically achieved through the recognition of specific target antigens displayed on the cancer cell surface. Once this binding occurs, it triggers a cascade of events within the NK cell, leading to its activation. Activated NK cells release cytotoxic granules, unleashing a lethal combination of factors that effectively dismantle the cancer cells, inducing apoptosis and eliminating the threat [8, 21]. TriKEs have demonstrated remarkable promise in a range of preclinical and early clinical studies across various cancer types. The design and optimization of TriKEs involve meticulous selection of target antigens to ensure specificity, fine-tuning of binding affinities to maximize their precision, and incorporation of immune cell activation domains to strengthen their cytotoxic potential. These considerations collectively contribute to shaping the overall efficacy and safety profile of TriKE-based therapies.

TriKEs stand as a testament to the remarkable progress in the field of cancer immunotherapy. Their unique tri-specificity, harnessing the capabilities of both NK cells and targeted cancer cell recognition, holds immense promise for more effective and safer cancer treatments. The ongoing research and development surrounding TriKEs continue to unveil new opportunities for innovation in the fight against cancer.

### Structures and designs of TriKEs

**Figure 1:**
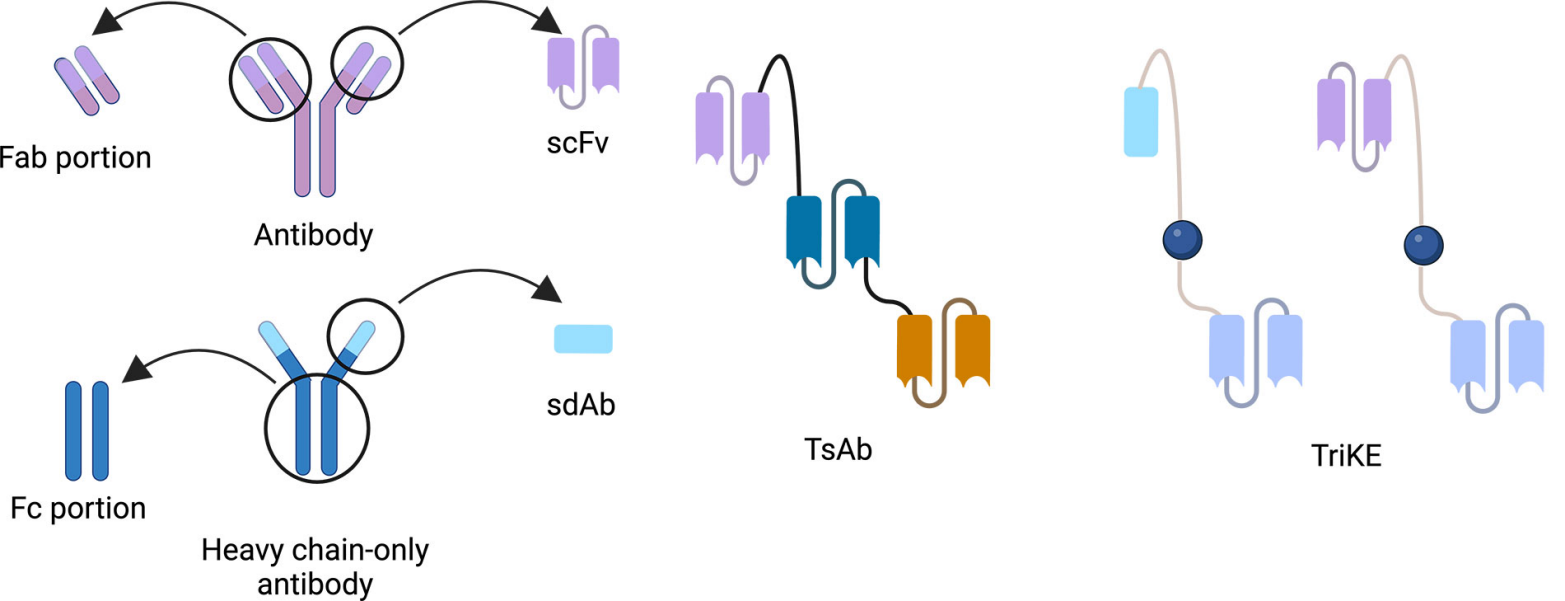
The correlative structure of antibody, TsAb and TriKE which are composed of scFv or sdAb, are linked together by a short amino acid sequence called flanking [22]. Created with BioRender.com

TriKEs are a combination of 3 distinct domains that are constructed from antigen-specific antibodies or part of antibodies (Figure 1). Formerly, TriKEs or other multi-specific immune cell engager were constructed by only scFv which consist of heavy chain and light chain variable domain which are connected with short amino acid sequences, called flanking [7, 13]. Lately, there have been a lot of formats to be used to construct. We can divide it into 2 groups of formats. One of them is immunoglobulin G (IgG)-like formats which include fragment crystallizable (Fc) region. Another is non-IgG-like formats which consists of antibody-derived building blocks without Fc region. So, the domain of non-IgG-like formats can be antigen-binding fragment (Fab) portion, scFv, or sdAb which have relatively smaller sizes than the IgG-like one with Fc portion [22, 23]. Each domain is also linked together by flanking [7].

The construction of TriKEs is composed of several combinations of antigen-specific antibodies or ligands of specific receptors. The most popular domain is composed of anti-CD16 and IL-15 which take roles in the activation of NK cells and enhancement of NK cell function and survival respectively. This review focuses on anti-CD16/IL-15 TriKE which is the most popular and effective combination of TriKE. While another part is cancer-specific and varies depending on the targeted cancer [7, 13–15].

In the early stages of TriKE development, the first generation of TriKEs employed anti-human CD16 scFv as a crucial component together with N72D modified human IL-15 superagonist where the asparagine at position 72 has been changed to aspartic acid [14, 24]. However, researchers encountered limitations in this configuration, primarily related to steric hindrance caused by the relatively large size of the two scFvs that directly affects to IL-15 accessibility, demonstrated with a function 25 times lower than that of recombinant human IL-15 [7, 25]. Recognizing the need for a more compact design, they embarked on a journey to streamline TriKE composition [20, 25]. To overcome this challenge, the research team innovatively introduced a humanized anti-CD16 heavy chain camelid sdAb, with a relatively smaller size, replacing the anti-human CD16 scFv. This strategic modification aimed to minimize steric hindrance and prevent undesirable pairing between multiple scFvs within the construct. The success of this innovation was remarkable, demonstrating prominently improved results. Notably, the newly incorporated anti-CD16 sdAb showcased superior efficacy in enhancing the impact of the IL-15 moiety. This achievement ensured that the use of mutated IL-15 within the construct was no longer necessary [22, 23, 25]. Building upon these notable findings, the second generation of TriKEs embraced the incorporation of anti-CD16 sdAbs, along with the critical presence of wild type IL-15 (wtIL-15). This strategic evolution marked a vital achievement in TriKE development, enhancing their effectiveness in targeting cancer cells and optimizing NK cell activation. For all previous studies of TriKE are listed in Table 1, specifying their format, specificity, tumor type, and target model.

**Table 1:** Previous studies of anti-CD16 × IL-15 TriKEs with different specificity in various cancer models

**Antibody format**	**Ligand specificity**	**Tumor type**	**Target model**	**References**
scFv-IL-scFv	CD33	Hematologic malignancy	AML	[7]
scFv-IL-scFv	EpCAM	Carcinomas	Carcinomas	[13]
scFv-IL-scFv	CD133	Solid tumor and hematologic malignancy	Colorectal carcinoma and Burkitt lymphoma	[14]
scFv-IL-scFv	CD19	Hematologic malignancy	B-CLL CD19^+^ tumor	[15]
scFv-IL-scFv	CD19	Hematologic malignancy	CD19^+^ tumor	[16]
sdAb-IL-scFv	B7-H3	Solid tumor	Ovarian cancer	[20]
sdAb-IL-scFv	CD33	Hematologic malignancy	AML	[25]
sdAb-IL-scFv	HER2	Solid tumor	Ovarian cancer	[26]
sdAb-IL-scFv	CLEC12A	Hematologic malignancy	AML	[9]
sdAb-IL-scFv	TEM8	Solid tumor	NSCLC	[10]
sdAb-IL-scFv	Mesothelin	Solid tumor	NSCLC	[27]
sdAb-IL-scFv	BCMA	Hematologic malignancy	MM	[11]

EpCAM: epithelial cell adhesion molecule; AML: acute myeloid leukemia; B-CLL: B cell chronic lymphocytic leukemia; NSCLC: non-small cell lung cancer; MM: multiple myeloma; HER2: human epidermal growth factor receptor 2; CLEC12A: C-type lectin domain family 12 member A; B7-H3: B7 homolog 3; BCMA: B-cell maturation antigen; TEM8: tumor endothelial marker 8

Moreover, there have been questioned that, instead of using TriKE including IL-15, induction of BiKE separately to IL-15 will not be better [14]. The reports from previously experiments confirmed that IL-15 in the TriKE construct has higher specificity to NK cell due to anti-CD16 which also improves preferable to NK cells instead of T cells. Demonstrated by higher in both lytic degranulation and interferon-γ (IFN-γ) secretion which are important signatures illustrated NK cell function. Besides, IL-15 specified to NK cell attenuates toxicity caused by undesired binding of IL-15 also [7, 14].

### TriKEs’ mechanism of action

NK cells are immune cell in innate immunity which take important roles in immunosurveillance and spontaneous cytolytic activity. NK cell function is driven by the balance of activating and inhibiting signal on the NK cell surface [21, 28, 29]. The signals are composed of various cytokines or antigens on the target cell surface those are normally different between host cells and malignant cells or foreign cells. The activating signals of NK cells can be stimulated through NK cell group 2 isoform C (NKG2C), NKG2D, DNAX accessory molecule-1 (DNAM-1), and NKp80, etc., by their ligands, such as IL-2, IL-15, IL-18, tumor necrosis factor-α (TNF-α), C-X-C motif chemokine ligand 10 (CXCL10) and IFN-γ. While the inhibiting receptors are composed of NKG2A, programmed cell death protein-1 (PD-1), killing Ig-like receptors (KIRs), and T cell immunoreceptor with Ig and ITIM domains (TIGIT), etc., stimulated through ligands, such as human leukocyte antigen (HLA) class I, IL-10, prostaglandin E2 (PGE2), transforming growth factor-β (TGF-β), and indoleamine 2,3-dioxygenase (IDO) [21, 29, 30].

**Figure 2:**
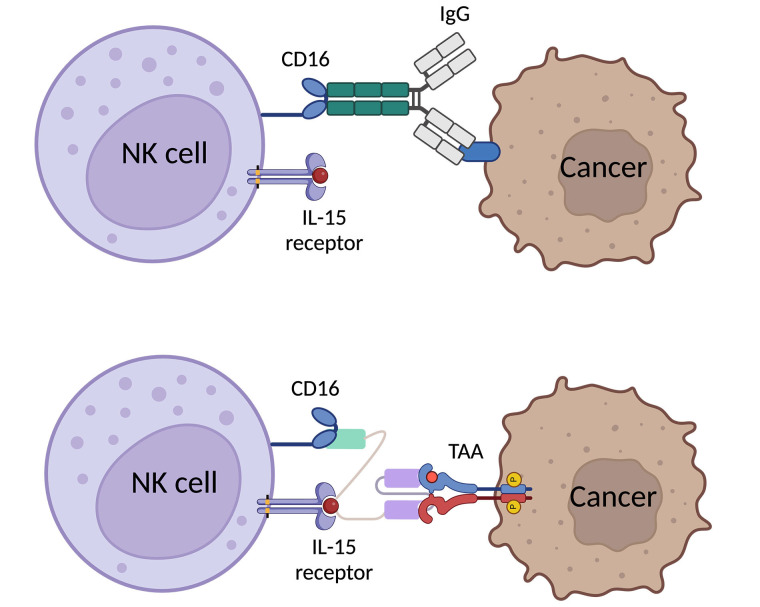
Graphic scheme of expected binding of TriKE to NK cell and cancer cell in comparative to natural binding of human IgG with Fc portion binding to CD16 [40, 41]. Created with BioRender.com

In malignant cells, the mutation alters the cell surface protein to express more activating ligands while losing some inhibiting ligands, resulting in NK cell activation and degranulation to kill those malicious cells [21, 30]. Once NK cells are activated, there are two main cascades to act against the abnormal cells. On one hand, NK cells exhibit CD107a-dependent functions which relate to the degranulation of NK cells. NK cells will secrete perforin and granzyme to induce apoptosis in target cells and inflammatory cytokines and chemokines to stimulate inflammation and recruit further immune cells, both innate and adaptive, to the site [31]. On the other hand, NK cells also perform CD107a-independent functions through the death receptor pathway, like the TNF-related apoptosis-inducing ligand (TRAIL) or fatty acid synthase ligand (FASL). Those receptors take a role in binding to their respective receptors or ligands on the target cells, ultimately inducing apoptosis [32, 33]. However, in some types of cancer, these cells express more inhibitory ligands, such as HLA, and/or exhibit reduced expression of activating ligands. Furthermore, certain tumor microenvironments (TMEs) can alter the expression of surface receptors on NK cells and secrete some factors, like IL-6, IL-10, PGE2, TGF-β [30], acids [34] and reactive oxygen species (ROS) [35] to prevent NK cell activation through both direct and indirect pathway [36, 37]. For example, TGF-β decrease the expression of activating receptors, NKp30 and NKG2D, while promoting regulatory T cells (Tregs) function. These effects result in the attenuation of NK cell function in TMEs. TriKEs can address these issues by efficiently activating NK cells through a highly effective pathway, called antibody-dependent cell cytotoxicity (ADCC) achieved through CD16 activation which is naturally stimulated by the Fc portion of IgG (Figure 2) [30, 36, 38, 39].

Additionally, those issues from TMEs can also diminish NK cell proliferation. To cope with this, previously, IL-2 had been used to enhance NK cell function in tumor suppression [42, 43]. But the researchers found that IL-2 also stimulate Tregs causing NK cell exhaustion, and potentially inducing vascular leak syndrome in mice *in vivo* studies [44]. Later, IL-15 was discovered to be a promise representative of IL-2 in NK cell immunotherapies with their similar function and processing through the same pathway. Importantly, IL-15 does not stimulate Treg but cytotoxic T cell and is not capable to induce vascular leak syndrome [8, 44–46]. In conclusion, TriKE incorporates IL-15 can deal with the immunosuppressive function of TMEs by promoting NK cell proliferation and persistence within TMEs excluding negative results in both NK cells and healthy cells. The last domain of TriKE is antigen-specific which takes a role in specifically target the cancer cells to complete their bridge function which connects enhanced NK cells to the targeted cancer cells. Previously, various types of specificity were designed, such as anti-CD33 AML [7, 25], anti-EpCAM against carcinomas [13], anti-CD19 against B-CLL [15] anti-TEM8 and anti-mesothelin against NSCLC [10, 27], anti-CLEC12A against AML [9], and anti-HER2 against ovarian cancer [26]. However, there are some interesting target antigens those have been used as potential target immunotherapy in other platforms, like monoclonal antibody (mAb), CAR-T cell, and other immune cell-engager: T cell engager and macrophage engager which can be applied to TriKEs construction [47].

### Clinical studies

Until now, there have been a lot of TriKE constructs that are progressing on *in vitro* studies (Table 1) [9–11, 13–16, 20, 25–27], but there is only one reached clinical studies. GTB-3550 TriKE which is a primal construct with CD16 scFv and targets on CD33^+^ AML and myelodysplastic syndromes (MDS) is the first TriKE in phase I clinical trials (NCT03214666) [7, 12]. Patients received treatment at doses of 5 µg/kg per day and 10 µg/kg per day, with two patients assigned to each dose group. The compromised treatment was observed with the dose-dependent manner of CD33^+^ blast clearance and NK cell proliferation and activation in AML and MDS patients. Furthermore, the short half-life was observed, illustrated the rapid clearance of TriKE without advance stimulation. Nevertheless, there were no observed instances of severe side effects [12]. Afterward, GTB-3550 was substituted by second-generation TriKE, GTB-3650, with camelids anti-CD16 sdAb which proved their superior affinity in binding to CD16 and stimulation of ADCC in preclinical studies [25]. Although the results in the preclinical stage are promising and have not shown any negative outcomes, further preclinical studies are still needed to validate the novel findings.

## Comparative analysis with other immunotherapies

### CAR-T and NK cell therapy

Chimeric antigen receptor (CAR) represents a groundbreaking genetic modification of immune cells, primarily T and NK cells, to express a CAR that is specific to tumor-associated antigens (TAAs) [48, 49]. In general, this recombinant protein consists of four different domains: the antigen recognition domain, the hinge region, a transmembrane domain, and an intracellular signaling domain. This specific feature of CAR offers the advantage of overriding cancer immune escape via major histocompatibility complex (MHC) downregulation. The recognition of CAR is MHC-independent, achieved by using the antigen recognition domain (i.e., single chain variable fragment and peptide) to directly target the tumor antigen on the cancer cell surface. The binding of antigen and CAR triggers the signaling domain to activate the anti-tumor activity, resulting in the cancer elimination. Consequently, it can redirect the specificity and improve the function of T and NK cells. Notably, adoptive cell transfer of genetically engineered cytotoxic immune cells armed with CAR holds greater promise for cancer treatment. This innovation has demonstrated remarkable potential as a cancer treatment, both *in vitro* and *in vivo*, and several types of CAR-T platform have obtained Food and Drug Administration (FDA) approval following successful clinical studies [50–54]. Currently, there are six FDA-approved CAR-T cells for blood cancer treatment: (1) CD19 specific CAR-T cells; tisagenlecleucel (Kymriah) for the treatment of pediatric and young adult acute lymphoblastic leukemia (ALL) in addition to axicabtagene ciloleucel (Yescarta), brexucabtagene autoleucel (Tecartus), and lisocabtagene maraleucel (Breyanzi) for the treatment of different B cell malignancies. (2) BCMA specific CAR-T cells; idecabtagene vicleucel (Abecma) and ciltacabtagene autoleucel (Carvykti) for the treatment of MM [55].

However, as CAR-T cell therapies have gained prominence, concerns have arisen regarding their adverse effects, prompting a closer examination of their specificity and potential toxicity [49]. One of the critical aspects of CAR-T cell therapy is its specificity. CARs are engineered to target particular cancer cells bearing associated antigens. However, this specificity extends not only to target cancer cells but also to non-target, normal cells that may express the same antigen, even at lower levels. This phenomenon can lead to off-tumor effects, where CAR-T cells inadvertently attack healthy tissues that share the targeted antigen with cancer cells. This off-tumor effect can have severe and harmful consequences [49, 56, 57]. Moreover, CAR-T cell therapy’s mechanism of action relies on the hyperactivation of T cells upon encountering the antigen, regardless of MHC receptor activation [48, 57]. While this can effectively target tumor cells, it can also result in excessive cytokine release and massive tumor necrosis. These processes can give rise to two notable complications. The first one is cytokine release syndrome (CRS) which can cause systemic inflammation, impacting various organs and leading to symptoms such as fever, fatigue, and even organ dysfunction [53, 54, 58–61]. Another one is tumor lysis syndrome (TLS) leading to electrolyte imbalances [56, 62, 63]. The underlying factor in these adverse outcomes is the hyperactivity of T cells.

In response to these challenges, there has been a growing interest in the development of NK cell-related immunotherapy approaches to mitigate these undesired effects. Unlike the CAR-T cells, CAR-NK cells have demonstrated promising outcomes with fewer side effects. NK cells employ an MHC-independent mechanism to recognize the cancer through the imbalance of activating and inhibitory molecules. This balance makes NK cells highly effective in distinguishing between normal and cancer cells [21, 28, 29] and subsequently activate the cytotoxicity by releasing lytic granule or inducing death receptor-mediated apoptosis through fatty acid synthase (FAS) or TRAIL cascade. In the CAR-NK platform, the CAR molecule, providing specificity and a distinct anti-tumor signaling cascade, greatly improves the cytotoxicity and specificity of NK cells. The MHC-independent mechanism of CAR-NK confers several advantages over CAR-T cells, including a low risk of graft-*versus*-host disease, CRS, and neurotoxicity, particularly, when compared to allogenic CAR-T cells.

However, the CAR platform utilizes engineered technology to embed the CAR molecule, which necessitates high-cost technology and is time consuming. Alternatively, TriKEs have emerged as promising strategies to redirect NK cells and limit complications related to T cell hyperactivity [8, 10, 64]. TriKEs with anti-CD16/IL-15/anti-TAA target NK cells as their effectors and can effectively modulate NK specificity and enhance NK cell function. As mentioned NK cell property earlier, NK cell activation via TriKEs presents a potentially safer pathway in immunotherapy with minimized cost and time production. Recombinant TriKEs provide as off-the-shelf products without the requirement of engineering process and autologous cells from patients resulting in more safety and highly promising. By harnessing the inherent specificity of NK cells and their ability to discern between healthy and cancerous cells, these approaches aim to reduce the risk of off-tumor effects and the complications associated with the hyperactivation of T cells. As the field of immunotherapy continues to evolve, the development and refinement of NK cell-based strategies hold great promise in improving the safety and efficacy of cancer treatments.

### Therapeutic vaccine

A therapeutic vaccine is a form of active immunization designed to manipulate anti-tumor activity. One of the most well-known therapeutic vaccines for cancer treatment is the dendritic cell (DC) vaccine. DCs function as professional antigen presenting cells (APCs), capable of processing antigens and presenting them via MHC I and MHC II to CD8^+^ and CD4^+^ T cells, respectively. DCs play a crucial role as a link between innate and adaptive immunity, activating both arms of the immune system, innate and adaptive immunity. DCs loaded with tumor antigen serve as an immunized vaccine, inducing antigen-specific cytotoxic T cells to recognize and eliminate cancer cells. Clinical trials in melanoma, prostate cancer, malignant glioma, and renal cell carcinoma have demonstrated safety without serious side effects, even in patients with advanced disease [65]. Unfortunately, while vaccine immunization has shown the induction of the specific anti-tumor immune response, the clinical response has been suboptimal and unsatisfactory. The objective tumor response rate has rarely exceeded 15% [65].

Due to the DC vaccine is a personalized approach that modulates the upstream of anti-tumor stimulation, its failure could be influenced by several factors. These may include the immunosuppressive environment, the absence of specific T cell clones, the presence of immune checkpoint proteins, or individual immune status. Furthermore, the production of a DC vaccine involves multiple steps based on the source of DC and faces limitations in mass production due to the difficulty of DC proliferation. In contrast, TriKEs technology offers a straightforward method to redirect the function of the NK population to specifically target and eliminate cancer cells. The *ex vivo* expansion of NK and their allogenic use overcome limitations in both the quality and quantity aspects of immune cell preparation compared to DC vaccines. Moreover, as off-the-shelf technology, TriKEs are more robust and can serve as a frontier immunotherapy. Given that TriKEs and DC vaccines promote different arms of immunity, their combination as therapy is plausible to enhance anti-tumor activity and provide the highest potential to combat cancer growth.

### Immune checkpoint inhibitors

Immune checkpoint inhibitors (ICIs) have revolutionized cancer immunotherapy by enhancing the activation of immune cells, particularly T cells, to improve their ability to target and kill cancer cells by blocking the regulatory checkpoint [66]. The main targets of ICIs composed of cytotoxic T-lymphocyte antigen-4 (CTLA-4) [67, 68] and PD-1/programmed cell death ligand-1 (PD-L1) [66]. Until now, there have been various agents based on ICI that were approved by the FDA and in clinical trials [66]. However, the clinical use of ICIs is associated with certain adverse effects, broadly called immune-related adverse events (irAEs), that warrant careful consideration [69, 70]. The irAEs can initially be characterized by two primary short-term adverse effects, which may eventually accumulate into more impactful long-term adverse effects or life-threatening [70, 71]. The first one is hyperactivation of T cells leading to on-target off-tumor effects. Several ICIs overstimulate T cells through blocking of main regulation pathway, resulting in damage to both malignant and non-malignant cells which can prolong to be hypophysitis, thyroiditis, pneumonitis, and autoimmune diabetes [70, 71]. Another one is tissue pathway inhibition. Some tissues in the body rely on immune checkpoint pathways for stability and regulation. When ICIs disrupt these pathways, it can lead to instability in these tissues, compromising their normal function. This disruption can give rise to autoimmune-like diseases, as ICIs inadvertently interfere with the tissue’s immune-regulatory mechanisms [70].

While ICIs have shown remarkable efficacy in cancer treatment, these adverse effects are remarkable concerns that need to be addressed. Here comes the role of TriKEs. TriKEs represent a promising advancement in immunotherapy that can address these adverse effects. Unlike ICIs, which primarily target immune checkpoints, TriKEs can be designed to target specific TAAs, bypassing the immune checkpoint altogether. The key advantage of TriKEs is their mechanism of action through NK cells [7]. TriKEs activate NK cells with high precision, inducing a targeted immune response against cancer cells without the same adverse effects associated with T cell hyperactivation seen in ICIs [8, 64]. NK cells are known for their lower propensity to cause cytokine storms and off-tumor effects, making them an attractive alternative for cancer immunotherapy [64]. In summary, while ICIs have notably improved cancer treatment, their adverse effects remain a challenge. TriKEs offer a promising solution by targeting specific TAAs through NK cell activation, mitigating the adverse effects seen with ICIs. Future research and clinical trials will further elucidate the potential of TriKEs in cancer therapy, offering hope for improved cancer treatments with fewer complications.

### Cytokine therapies

Cytokines serve as central regulators of the immune system, acting as messengers between immune cells and exerting control over various cellular functions, including the induction of inflammation and apoptosis in malignant cells [47]. Over the years, several cytokines have received approval for use in human therapies, offering promising avenues for cancer treatment. These include IFN-α, IFN-γ, IL-2, IL-12, IL-15, IL-21, and granulocyte-macrophage colony-stimulating factor (GM-CSF), among others [42, 43, 47]. Nonetheless, it’s important to acknowledge that cytokine therapies come with their set of difficulties. Even though these therapies have proven effective in enhancing immune responses against cancer, they have also been linked to various side effects, including some that can be severe and potentially life-threatening [43, 46, 71, 72]. For instance, IFN-α, employed in melanoma patients [47], has been linked to flu-like symptoms, hematological toxicity, elevated liver enzyme levels, nausea, fatigue, and psychiatric sequelae. These side effects often correlate with the dose administered, making high-dose treatments particularly challenging [73]. Similarly, IL-2, used in metastatic renal cancer and melanoma [47], can lead to a life-threatening condition known as vascular-leak syndrome [46]. Given the limitations associated with high-dose cytokine therapy due to its potential for severe adverse effects, it is increasingly recognized as a complementary treatment to be combined with other primary therapies [47, 72]. The question arises as to whether low-dose cytokine therapy possesses sufficient potential to combat cancer effectively [47]. TriKEs offer a promising solution that bridges the gap between cytokine therapy and targeted immunotherapy [7]. These innovative molecules incorporate one IL-15 domain, a cytokine known for its role in stimulating NK cell proliferation and prolonging their function. In addition to their cytokine activity, TriKEs activate NK cells through CD16 engagement and target cancer cells through a third TAA-specific domain [7–11]. This unique combination of features positions TriKEs as a versatile tool in cancer immunotherapy. By harnessing the benefits of cytokine stimulation while precisely directing immune cell activity, TriKEs aim to provide a more focused and effective approach to combating cancer [8, 23, 64, 74]. Moreover, their ability to engage NK cells in a highly specific manner reduces the risk of off-target effects and severe adverse reactions, offering a more balanced and safer immunotherapeutic option [64]. In summary, while cytokine therapy has made prominent contributions to cancer treatment, it comes with challenges and limitations, necessitating careful consideration in clinical practice. TriKEs represent an innovative approach that combines the benefits of cytokine stimulation with targeted immunotherapy, potentially offering a more effective and safer solution for cancer patients.

### mAbs

mAbs are precision-engineered immunotherapeutic agents used widely in modern medicine. They are designed to target specific antigens, making them valuable in various fields, including cancer treatment [75, 76]. The mAbs have a dual structure, comprising the Fab region for antigen recognition and the constant Fc region for interacting with the immune system [76]. The Fc region plays a crucial role in stimulating immune cell responses [77]. While mAbs show remarkable therapeutic potential, they have factors to consider, including their size, persistence, and potential for unintended immune cell activation, leading to off-target effects and complications [76].

TriKEs were initially based on the function of antibodies, including mAbs [2]. However, their construct has been influentially altered to enhance their effectiveness beyond traditional antibodies [41]. TriKEs, with their compact size due to the absence of the Fc portion [7, 25], facilitate efficient biodistribution, allowing them to navigate through the circulatory system and reach target tissues, including solid tumors [41]. This advantage becomes particularly pronounced in the challenging microenvironments of solid tumors, where TriKEs can penetrate barriers effectively [20]. In contrast, the larger size of mAbs can hinder their distribution within solid tumors, potentially limiting their therapeutic reach [78]. However, the smaller size of TriKEs also means a shorter half-life, requiring careful dosing strategies [41].

In summary, mAbs and TriKEs have distinct characteristics, with TriKEs offering advantages in biodistribution due to their smaller size and altered construct. This property enhances their potential as a valuable tool in cancer immunotherapy, striking a balance between size-related advantages and therapeutic effectiveness. Some functions and adverse effects of those immunotherapies are summarized in Table 2.

**Table 2:** Comparison of each immunotherapy in terms of major effector, limitation, and undesired adverse effects

**Function**	**TriKEs**	**CAR-T cells**	**ICIs**	**CKTs**	**mAbs**
Functions					
Major effector cells	NK cells	T cells	T cells	Various immune cells	Various immune cells
Limitation	Frequent dosing requirement	Excessive immune response	Excessive immune response	Effective dose’s cytotoxicity	Varied effects among people
	Antigen escape	High costs	Natural pathway inhibition	Uncertain stimulation	Limited penetration accessibility
Adverse effects					
Stimulation of CRS	No reports	Anti-CD19 CAR in B-CLL and DLBCL patients [53, 54, 61]	Anti-PD-1/PD-L1 in melanoma and hemotologicmalignancy [79]	No reports	Anti-CD3, anti-CD20, and anti-CD28 [58, 75]
On-target off-tumor cytotoxicity	No reports	Anti-TAG72, anti-B7-H3 [56, 57]	Anti-PD-1 and anti-CTLA-4 [70]	No reports	Anti-TNF and anti-EGFR [75]
Neurotoxicity	No reports	Anti-CD19 CAR in B-CLL patients [53, 56]	Anti-CTLA-4 and anti-PD-1 [80]	No reports	No reports
Graft-*versus*-host disease	No reports	Anti-CD19 CAR in B-CLL [53]	Anti-PD-1 and anti-CTLA-4 [70]	No reports	No reports
Others	No reports	TLS [56]	Autoim-mune diabetes [70]	Vascular-leak syndrome [44]	No reports

CKTs: cytokine therapies; TAG: tumor-associated glycoprotein; DLBCL: diffuse large B-cell lymphoma; EGFR: epidermal growth factor receptor

## The future direction of TriKEs

As it is looked ahead to the future of TriKEs, these innovative immunotherapies hold great promise in the realm of cancer treatment. However, like any evolving medical technology, there are challenges and disadvantages that researchers are actively working to address [11, 27]. In this section, we will explore some of the key considerations and strategies for overcoming these drawbacks, with a focus on optimizing the function of TriKEs while attenuating the risks of off-target effects.

One primary challenge of TriKE, addressing their compact design challenge is crucial. Their smaller size and shorter half-life demand more frequent dosing [41], which could pose logistical and cost concerns. Thus, research focuses on innovative dosing and delivery for convenience and cost-effectiveness [41]. On the other hand, TriKEs’ compactness offers precise immune modulation to prevent hyperactivation, crucial for safety [81]. Although there is no head-to-head comparison of possible side effects of TriKEs and related bi-specific T cell engagers (BiTEs), lessons from CAR technology, comparing CAR-T cells and CAR-NK, suggest a lower risk of side effects with NK than with T cells. CRS and a neurologic event have been reported in the use of blinatumomab, an FDA-approved BiTE specific to CD19 for the treatment of advanced ALL [82, 83]. On the other hand, a clinical trial of TriKE specific to CD33 (NCT03214666) for treatment of relapse/refractory AML reported completed therapy (5–150 μg/kg per day) without dose limiting toxicity [12], suggesting the advantage of TriKEs. Currently, ongoing studies aim to refine this balance for potent yet safe TriKE therapies. Researchers explore combining TriKEs with other treatments to maximize impact and reduce the need for higher doses.

TAA expression levels and heterogeneity present ongoing challenges in cancer treatment. Some antigens may be present in both cancer and healthy cells, necessitating precise TriKE design for specificity [84]. Studies explore the induction of tumor-specific antigens (TSAs), commonly referred to as neoantigens, to enhance treatment specificity while minimizing harm to healthy cells. In addition to meticulously selecting appropriate TAA, neoantigen therapies have been combined with adoptive cell therapy [85], mAbs [86], or bi-specific antibody (BsAb) [85] to mitigate damage to healthy cells due to off-target effects.

Furthermore, there is a clue of upregulating existing antigens to enhance tumor cell sensitivity, especially in conjunction with BiTEs, holds promise in cancer treatment [87]. These advancements enhance selectivity, minimizing harm to healthy cells while maximizing their impact on cancer cells. These compelling strategies represent a potent approach for their application in enhancing the effectiveness of TriKEs against cancer. Additionally, Vallera and his team [84] have investigated dual tumor targeting, known as tetraspecific killer engager (TetraKE) with two TAAs, to address tumor heterogeneity and simultaneously target both carcinoma and cancer stem cells [9]. TetraKE was designed to prevent the emergence of antigen-negative cells after TriKE treatment, as the elimination of tumor cells with one specific antigen may leave antigen-negative cells behind.

Another problem that NK cell-based immunotherapy has to face is CD16 downregulate in some cancer patients especially in solid tumors [20, 26] and TMEs [88]. It’s still unclear about their exact mechanism, the existed results suggested the downregulation of CD16 on activated NK cell [20, 27]. Another avenue for future exploration in TriKE-based therapies is the combination with A disintegrin and metalloprotease 17 (ADAM17) inhibition [77, 89, 90]. ADAM17, a surface protein on NK cells, plays a role in modulating immune responses. By inhibiting ADAM17 alongside TriKE treatment, researchers aim to enhance the therapeutic effects of TriKEs while concurrently upregulating IFN-γ, a critical player in the anti-cancer immune response [89]. This dual action holds the potential to improve the overall efficacy of TriKE-based therapies [9, 77, 89]. Furthermore, ongoing research into TriKEs could focus on identifying and harnessing new activating receptors of NK cells, such as NKG2D [91] or NKp46 [92]. Expanding the repertoire of immune targets broadens the applicability of TriKEs and their potential to address various cancer types and scenarios. Additionally, exploring other TAAs previously targeted by platforms like BiTE or mAbs offers exciting opportunities for TriKE-based immunotherapies. Previous BiTE, BiKE, and TriKE have also been applied from former mAbs or ICIs, like CD3 × PD-L1 BiTE [69, 87], CD16 × CD33 BiKE [93, 94] or CD133 TriKE [14, 95]. There are numerous targets to be applied to TriKE construct to sensitize NK cell to cancer cells, such as CD22 on ALL, PD-L1 on bladder cancer, NSCLC and various carcinoma, signaling lymphocyte activation molecule family 7 (SLAMF7) on MM, vascular endothelial growth factor receptor-2 (VEGFR-2) on gastric cancer [40, 75], CD47 on non-Hodgkin lymphoma and several carcinomas [96]. Some studies have explored the synergistic combination of BiKEs with CAR-NK therapy. This approach not only activates NK cells and targets cancer through CD16 but also engages downstream pathways through CAR platforms [97]. The synergy between these two immunotherapeutic approaches offers a multifaceted strategy for enhancing anti-cancer immunity, noted that can apply to TriKE. The future of cancer treatment may involve integrating these complementary approaches for more effective and adaptable solutions.

In summary, the future directions of TriKEs in cancer immunotherapy encompass several exciting avenues of research and development. These strategies collectively aim to advance the field of cancer immunotherapy, offering more precise, effective, and adaptable solutions for treating a range of cancers.

## Conclusions

TriKEs are at the forefront of innovation in cancer immunotherapy, ready for in-depth research and widespread adoption. These remarkable agents exhibit a multifaceted approach by concurrently activating and sustaining the activity of NK cells while specifically targeting cancer cells. The result is a potent and multifunctional strategy against malignancies. Through iterative refinements and innovation, TriKEs have evolved into highly effective immunotherapies for cancer treatment. They stand out for their ability to offer a range of benefits, including minimal complications, cost-effectiveness, rapid development timelines, independence from genetic modifications or adoptive cell therapies, off-the-shelf availability, and ability to cope with others’ weaknesses. Despite some identified limitations, the overall efficiency of TriKEs surpasses these challenges. The versatility of TriKEs is a key asset, as they can be tailored to combat a wide spectrum of cancer types. Their applicability extends to various scenarios, making their integration into cancer treatment strategies both promising and accessible. In summary, TriKEs hold great promise as a transformative force in the field of cancer immunotherapy. With their remarkable attributes, they offer a multifaceted and effective approach to combating cancer, paving the way for novel and accessible treatment options across diverse cancer diseases.

## Abbreviations

ADAM17: A disintegrin and metalloprotease 17
ALL: acute lymphoblastic leukemia
AML: acute myeloid leukemia
B7-H3: B7 homolog 3
B-CLL: B cell chronic lymphocytic leukemia
BCMA: B-cell maturation antigen
BiKE: bi-specific killer engager
BiTEs: bi-specific T cell engagers
CAR: chimeric antigen receptor
CAR-T: chimeric antigen receptor-T
CD16: cluster of differentiation 16
CLEC12A: C-type lectin domain family 12 member A
CRS: cytokine release syndrome
CTLA-4: cytotoxic T-lymphocyte antigen-4
DC: dendritic cell
EpCAM: epithelial cell adhesion molecule
Fc: fragment crystallizable
FDA: Food and Drug Administration
HER2: human epidermal growth factor receptor 2
ICIs: immune checkpoint inhibitors
IFN-γ: interferon-γ
IgG: immunoglobulin G
IL-15: interleukin-15
mAb: monoclonal antibody
MHC: major histocompatibility complex
MM: multiple myeloma
NK: natural killer
NKG2C: natural killer cell group 2 isoform C
NSCLC: non-small cell lung cancer
PD-1: programmed cell death protein-1
PD-L1: programmed cell death ligand-1
scFvs: single-chain fragment variables
sdAbs: single-domain antibodies
TAAs: tumor-associated antigens
TEM8: tumor endothelial marker 8
TGF-β: transforming growth factor-β
TMEs: tumor microenvironments
TNF-α: tumor necrosis factor-α
Tregs: regulatory T cells
TriKEs: tri-specific killer engagers

## Declarations

### Copyright

© The Author(s) 2024.
